# Consanguinity and adverse fetal outcomes- a population-based cohort study from a multiethnic population in the Middle East

**DOI:** 10.1186/s12884-025-08308-2

**Published:** 2025-12-17

**Authors:** Jis Thomas, Fathima Minisha, Najat Khenyab, Salwa Abuyaqoub, Husam Salama, Sawsan Al Obaidly, Nader Aldewik, Hilal AlRifai, Thomas Farrell

**Affiliations:** 1 Department of Obstetrics and Gynaecology, Women’s Wellness and Research Centre, Doha, Qatar; 2 College of Medicine, Qatar University, Doha, Qatar; 3 Department of Pediatrics and Neonatology, Women’s Wellness and Research Centre, Hamad Medical Corporation, Doha, Qatar; 4 Department of Research, Women’ s Wellness and Research Centre, Doha, Qatar; 5 Chief Executive Officer, Women’s Wellness and Research Centre, Hamad Medical Corporation, Doha, Qatar

**Keywords:** Consanguinity [MeSH], Consanguineous unions, Congenital anomalies, Stillbirth, Neonatal death, Reproductive loss

## Abstract

**Background:**

Consanguinity occurs widely in the Middle East and is associated with reproductive loss, congenital malformations, metabolic disorders, and autosomal recessive disorders. Qatar has a high consanguinity rate; however, large-scale population-based studies evaluating perinatal outcomes are lacking.

**Methods:**

A retrospective cohort study of pregnancies ≥ 20 weeks of gestational age in Qatar from a maternity registry was conducted. Women were divided into consanguineous unions (CU) and non-consanguineous unions (NCU) based on their relationship with their spouses. The CUs were further divided into first-degree and second-degree. The outcomes included congenital anomalies, chromosomal anomalies, preterm births, stillbirths, neonatal deaths and small for gestational age (SGA).

**Results:**

The rate of consanguinity among the women in the cohort was 30.8% (46.4% among Qatari women), with higher rates of first-degree (74%). Younger maternal age, obesity, higher parity and lower maternal education were factors associated with CUs. The incidences of anomalies, stillbirth and neonatal deaths in CUs were 21, 4.3 and 2.4 per 1000 births, respectively. After adjusting for confounders, CUs had higher odds of major congenital anomalies (aOR = 1.37; *p* = 0.049), stillbirth (aOR = 3.10, *p* = 0.007) and neonatal deaths (aOR = 3.15, *p* = 0.042) in non-anomalous babies, the difference being larger in non-Qatari women. Cardiovascular anomalies were most common, higher in first-degree CU, with nervous system anomalies higher in expatriates.

**Conclusions:**

This study concludes that consanguinity is associated with an array of major risks, including congenital anomalies, stillbirths, and neonatal deaths, even in morphologically normal babies. The results are intended to raise awareness about the consequences of CUs in the country, in order to improve the genetic constitution and long-term health of future generations.

## Introduction

Consanguinity, or a consanguineous union (CU), is defined as a marital relationship between individuals who have descended from the same ancestors, more specifically between second cousins or closer as per clinical genetics [1]. The current global estimate of consanguinity is about 10.4%, with some parts of the world, including the Middle East, exhibiting higher numbers, reaching upto 50% [2]. The factors determining the rates of consanguinity in a population are diverse, including socioeconomic, political, religious, and cultural factors.

There is a wealth of medical literature emphasising the deleterious effects of parental consanguinity on reproductive outcomes. The sharing of genetic material results in homozygosity in the offspring of CUs and an increase in autosomal recessive disorders. Globally, various studies report a higher risk of congenital anomalies, stillbirths, neonatal and infant deaths in CU babies compared to the non-consanguineous unions (NCU) [3–5]. Studies on women from areas in the Middle East such as Saudi Arabia, Kuwait, Omar and Lebanon demonstrate an association between first-degree consanguinity and congenital anomalies, more specifically, cardiovascular anomalies [6–9], although there are conflicting reports regarding associations with stillbirths and infant mortality.

The rate of CUs has increased in Qatar over the past generation, estimated to be as high as 54% in the nationals, with an average coefficient of inbreeding being 0·02706 (or 2.7%), considering both first-degree and second-degree CUs [10, 11]. The commonest type is first-degree CUs (nearly 35% of all marriages), referring to only first-cousin marriages since CUs among individuals more closely related are legally not permitted in the country. The higher rate could be attributed to a lack of knowledge regarding the impact of consanguinity on autosomal recessive diseases and congenital anomalies in the offspring and the genetic risks associated with different levels of CUs [12]. Studies report higher odds of autosomal recessive diseases, congenital anomalies and hereditary hearing loss in offspring of CU in Qatari women [13, 14]. However, Qatar has a diverse expatriate population from countries where CUs are customary, with the nationals representing only a third of the women delivering in the country. There is a lack of studies evaluating the impact of CUs on perinatal outcomes in the multiethnic population in Qatar, as previous reports have focussed mainly on the nationals.

The rising trend in CUs and the potential risks to the offspring make it imperative to address the knowledge gap caused by the lack of large-scale studies from Qatar. The primary aim of this study is to compare the perinatal outcomes between pregnancies from CUs and NCUs in the multiethnic population in the country, using a large maternal registry data. The findings of the study can be used to raise awareness about CUs and improve the screening and surveillance of these high-risk pregnancies in order to improve the health of future generations in Qatar.

## Methods

### Setting and participants

The sample for this population-based retrospective cohort study was selected from all women delivering in Qatar between January 2017 and April 2018, included in the perinatal maternity registry. The study was held at Women’s Wellness and Research Centre, the largest tertiary care maternity centre in the country, accounting for the largest proportion of births in Qatar. Ethical approval was obtained from the Medical Research Centre, Hamad Medical Corporation (MRC-01–24−047), with a waiver of written consent as only data in the maternity registry collected from patient electronic health records were used in the analysis.

All the pregnancies with gestational age (GA) ≥ 20 weeks at the time of delivery were considered for inclusion. Women with information regarding consanguinity available and with documented antenatal care in the government primary, secondary or tertiary care centres in Qatar were included. They were divided into consanguineous unions (CU) and non-consanguineous unions (NCU). The CU group were further divided into first-degree, defined as unions between individuals who share the same second-last preceding generation or share less than 12.5% of their DNA (for example- first cousins), and second-degree, defined as unions between individuals who share less than 6.5% of their genes (for example 2nd cousins) [15]. Since unions between individuals sharing a preceding generation (such as siblings, uncles, aunts, nieces and nephews) are not legally permitted in the country, no such unions were present in this study. Women with missing data in any demographic or outcome variables were excluded, and a complete case analysis was done.

### Variables

All data was extracted anonymised from the maternity registry, which contained data obtained by independent data collectors from patients’ electronic medical records. Maternal demographic variables included maternal age in years, maternal body mass index (BMI) in kg/m^2^, parity- defined as any previous birth of > 24 weeks gestation, divided into nulliparous (parity = 0), multiparous (parity 1–3) and grand multiparous (parity ≥ 4), maternal nationality, maternal education divided into uneducated/elementary school, secondary/high school, and university, use of assisted reproduction techniques (ART), preexisting comorbidities such as diabetes, hypertension, thyroid disease, anemia, thrombophilia and cardiovascular disease, and location of delivery- in tertiary care or in peripheral secondary care.

The pregnancy outcomes included variables such as multiple gestation or higher-order pregnancies- defined as more than one fetus in the same pregnancy, gestational diabetes (GDM) defined by an abnormal glucose tolerance test performed in the second trimester, pregnancy-induced hypertension (PIH)- defined as new-onset of hypertension after 20 weeks gestation including the preeclampsia spectrum, and preterm birth (PTB)- defined as gestational age (GA) at birth less than 37 completed weeks. Additionally, PTB less than 34 completed weeks GA were also evaluated.

The fetal and neonatal outcomes considered were congenital anomalies- defined as structural or functional abnormalities including metabolic disorders present at birth, chromosomal anomalies such as trisomies diagnosed antenatally or postnatally, stillbirth defined as a baby delivered at ≥ 24 weeks GA showing no signs of life [16], neonatal deaths (ND)- defined as death occurring within 28 days of life in viable live-born babies (≥ 24 weeks GA) as per the World Health Organization (WHO) [17], small for gestational age fetus (SGA)- defined as a fetus with estimated fetal weight or abdominal circumference < 10th centile as per Hadlock’s centiles [18] in third-trimester ultrasound scans, low birth weight (LBW)- defined as birthweight < 2500 g as defined by WHO [19], and LBW in babies born at term (≥ 37 completed weeks GA) was evaluated in this study.

Variables associated with the exposure and outcome, such as age, BMI, parity, use of ART and preexisting comorbidities were considered for inclusion in the model as confounders. Maternal education is strongly associated with the exposure but not with the outcomes such as fetal anomalies and fetal death. This variable is reflective of the cultural preferences of families where early marriages in CUs are given a higher priority over completing education, especially among the nationals. Hence, we did not adjust for maternal education as it’s a strong predictor of the exposure.

### Data analysis

Continuous variables were represented as means and standard deviations (SD) or median and interquartile range (IQR), according to the distribution of the variable determined by analysing histograms and using Shapiro Wilk test. Categorical variables are represented using frequencies and percentages of the total. The demographics were compared between CU and NCU groups using the Student t-test or Wilcoxon ranksum test and Chi-square or Fisher’s exact test as appropriate.

The association between the outcomes and consanguinity were analysed using univariate and multivariable logistic regression models, giving crude odds ratios (ORs) with 95% confidence intervals (CIs) and adjusted ORs (aOR) after accounting for confounding variables not in the causal pathway. The NCU group was considered the baseline. The missing data in the variables were automatically excluded in the adjusted models. For outcomes such as preterm birth, LBW, SGA, stillbirth, and neonatal birth, only pregnancies ≥ 24 weeks were analysed (*N* = 10,874). The type of congenital anomaly (central nervous system- CNS, cardiovascular- CVS, and renal) was compared between first-degree CU, second-degree CU and NCUs, and the proportions were represented using bar graphs in Qatari and non-Qatari women. A p-value < 0.05 was considered strong evidence against the null hypothesis of no difference and of statistical significance. All analyses was performed using Stata statistical software, Release 18 (College Station, TX: StataCorp LLC).

## Results

**Fig. 1:**
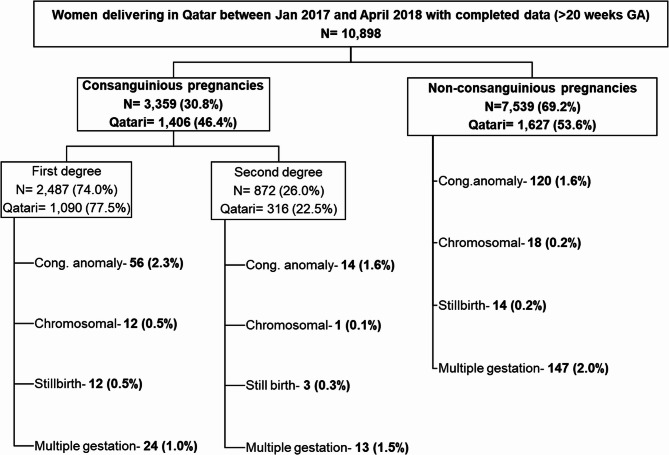
Participant distribution and exposure groups; Cong- congenital

Among 10,898 women included in this study, 30.8% (*N* = 3359) belonged to the CU group compared to 69.2% (*N* = 7539) in the NCU group. 74% (*N* = 2,487) of women in the CU group had first-degree consanguinity, while the remaining had second-degree (26%, *N* = 872), as shown in Fig. 1.

### Maternal demographics

The mean age of the cohort was 29.5 years (SD 5.4), with women in CU statistically significantly younger than in NCU (28.7 ± 5.7 vs. 29.9 ± 5.2, *p* < 0.001), as shown in Table 1. More than 50% of the cohort belonged to the obese category, which was similar across the comparison group, although those in CU had a significantly higher BMI at delivery. The median parity in CU was 2 (IQR 1–3) compared to 1(IQR 0–2) in NCU, with grand multiparity significantly more in the CU group (20.1% vs. 10.3%, *p* < 0.001). Similarly, 41.9% of the CU group were Qatari nationals compared to only 21.6% in the NCU group (*p* < 0.001). The majority of non-Qatari women were from other Arab countries in the Middle East (41% of total) and South Asia (20% of total). 46% of the Qatari women had a CU compared to 25% in the non-Qatari women. A significantly higher proportion of women in CU has a lower educational level, with more than 16% being uneducated or only attending elementary school compared to 5.6% in NCU (*p* < 0.001).

**Table 1:** Maternal demographics in the comparison groups

Variables		Total *N* = 10,898	Consanguinity		*P* value
			Yes; CU (*N* = 3,359)	No; NCU (*N* = 7,539)	
Maternal age in years; Mean (SD)		29.5 (5.4)	28.7 (5.7)	29.9 (5.2)	< 0.001
BMI categories; n (%N) Missing 70	< 25 kg/m^2^	1,258 (11.6%)	412 (12.4%)	846 (11.3%)	< 0.001
	25–30 (Overweight)	3,637 (33.6%)	1,045 (31.3%)	2,592 (34.6%)	
	30–35 (Obese I)	3,488 (32.2%)	1,039 (31.1%)	2,449 (32.7%)	
	≥ 35 (Obese II, III)	2,445 (22.6%)	841 (25.2%)	1,604 (21.4%)	
Parity; Median (IQR)		1 (0–3)	2 (1–3)	1 (0–2)	< 0.001
Parity categories; n (%N)	Nulliparous	2,933 (26.9%)	767 (22.8%)	2,166 (28.7%)	< 0.001
	Multiparous	6,513 (59.8%)	1,917 (57.1%)	4,596 (61.0%)	
	Grand multiparous	1,452 (13.3%)	675 (20.1%)	777 (10.3%)	
Qatari Nationality; n (%N)		3,033 (27.8%)	1,406 (41.9%)	1,627 (21.6%)	< 0.001
Education level Missing 1193 (11%)	Uneducated/Elementary school	846 (8.7%)	460 (16.4%)	386 (5.6%)	< 0.001
	Secondary/High school	3,150 (32.5%)	1,149 (40.9%)	2,001 (29.0%)	
	University	5,709 (58.8%)	1,199 (42.7%)	4,510 (65.4%)	
Assisted reproduction; n (%N)		318 (2.9%)	96 (2.9%)	222 (2.9%)	0.853
Location of delivery; n (%N)	WWRC	8,722 (80.1%)	2,917 (86.9%)	5,805 (77.0%)	< 0.001
	Periphery	2,171 (19.9%)	441 (13.1%)	1,730 (23.0%)	
Preexisting diabetes; n (%N)		263 (2.4%)	80 (2.4%)	183 (2.4%)	0.946
Chronic hypertension; n (%N)		101 (0.9%)	21 (0.6%)	80 (1.1%)	0.030
Thyroid disease; n (%N)		934 (8.6%)	293 (8.7%)	641 (8.5%)	0.711
Anemia; n (%N)		1,280 (11.7%)	496 (14.8%)	784 (10.4%)	< 0.001
Asthma; n (%N)		248 (2.3%)	92 (2.7%)	156 (2.1%)	0.037
Thrombophilia; n (%N)		35 (0.3%)	12 (0.4%)	23 (0.3%)	0.714
Cardiovascular disease; n (%N)		41 (0.4%)	13 (0.4%)	28 (0.4%)	0.867
No preexisting medical; n (%N)		5,729 (52.6%)	1,736 (51.7%)	3,993 (53.0%)	0.220

CU- consanguineous unions; NCU- non-consanguineous union; SD- standard deviation; BMI- body mass index; continuous variables compared using Students ttest or Wilcoxon ranksum test; Categorical variables compared using Chi-Square/Fishers exact; *p* < 0.05 considered evidence against null hypothesis of no difference between groups

More than 80% of women in the cohort chose the tertiary centre over other peripheral centres for delivery. This preference was more striking for women in CU (86.9%) compared to NCU (77.0%). Nearly 3% of women in both groups conceived via assisted reproduction techniques. Almost 12% of women suffered from anemia, the proportion increasing to 14.8% in CU compared to 10.4% in NCU (*p* < 0.001). Nearly 1% had preexisting chronic hypertension, which was similar across the groups (0.6% in CU vs. 1.1% in NCU). More than 50% of the cohort had no comorbidity (similar across both groups), with no significant difference noted for comorbidities such as preexisting diabetes, thyroid disease, asthma, thrombophilia, and cardiovascular disease.

### Pregnancy and neonatal outcomes

**Fig. 2:**
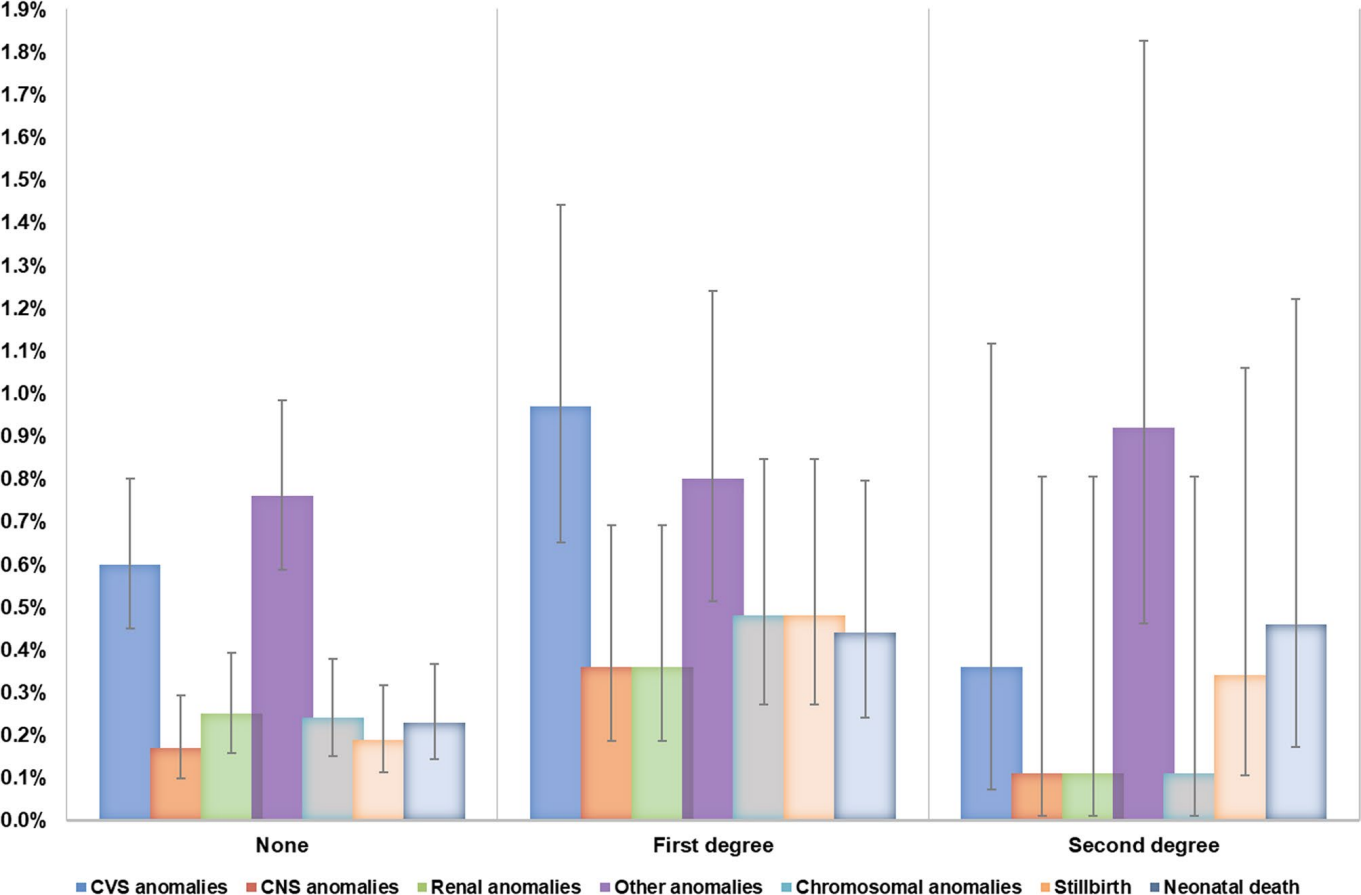
Proportion of outcomes comparing consanguinity first and second degree with no consanguinity (proportions are rounded off to the nearest one digit; error bars represent 95% confidence intervals)

**Fig. 3:**
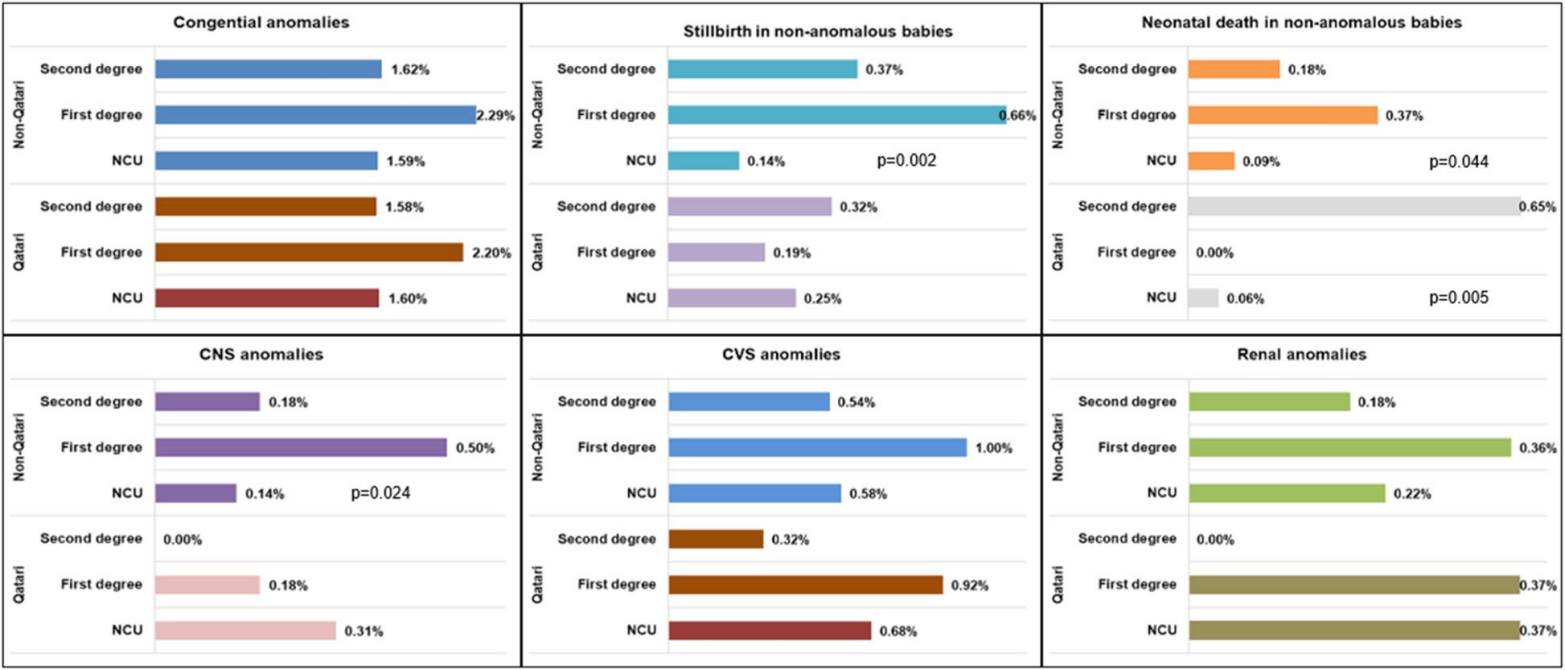
Neonatal outcomes in consanguineous unions vs non-consanguineous unions, divided based on nationality

Table 2 shows the perinatal outcomes in the exposure groups, with the crude and adjusted ORs. More than 1.7% of the cohort had babies with major congenital anomalies, the odds being 37% higher in CU (aOR = 1.37, 95% CI = 1.00–1.86.00.86; *p* = 0.049) compared to NCU. The incidence was 23 per 1000 births in those with first-degree consanguinity compared to 16 per 1000 births in those with second-degree. The incidence of major system anomalies is shown in Figs. 2 and 3. First-degree consanguinity was strikingly associated with higher incidence of CVS, CNS and renal anomalies when compared to second-degree and NCU. The CNS anomalies were strikingly more in the first-degree CUs among non-Qatari women (5 per 1000 versus 1.4 per 1000; *p* = 0.024), whereas for CVS and renal anomalies, the difference between CU and NCU, although more pronounced in non-Qatari women, were not statistically significant. The differences in the incidences of stillbirth in non-Qatari women were statistically significant (1 per 1000 in CU versus 0.1 per 1000 in NCU, *p* = 0.002), which contrasted remarkably with the Qatari women. However, for neonatal death, the differences between CU and NCU were statistically significant for both nationality groups, strikingly more in Qatari, as shown in Fig. 3.

**Table 2:** Crude and adjusted Odds ratios for the pregnancy outcomes

Pregnancy outcomes (total number in adjusted models) Total *N* = 10,898	Consanguinity (Baseline- NCU)		Crude OR (95% CI)	*P* value	Adjusted OR (95%CI)	*P* value
	Yes; CU (*N* = 3,359)	No; NCU (*N* = 7,539)				
Gestational diabetes (*N* = 10,566)	875 (26.7%)	2,069 (28.1%)	0.93 (0.85–1.02)	0.125	1.05 (0.95–1.16)	0.313
Gestational hypertension (*N* = 10,727)	74 (2.2%)	204 (2.7%)	0.81 (0.62–10.6)	0.117	0.97 (0.73–1.29)	0.843
Multiple gestation (*N* = 10,828)	37 (1.1%)	147 (2.0%)	0.56 (0.39–0.81)	0.002	0.47 (0.31–0.71)	< 0.001
#Preterm birth (< 37 weeks) (*N* = 10,804)	571 (17.0%)	1,302 (17.3%)	0.98 (0.88–1.09)	0.712	0.94 (0.84–1.06)	0.313
#Preterm birth (< 34 weeks) (*N* = 10,804)	55 (1.6%)	140 (1.9%)	0.88 (0.64–1.20)	0.421	0.95 (0.68–1.32)	0.751
#Low birth weight (*N* = 10,804)	265 (7.9%)	558 (7.4%)	1.07 (0.92–1.25)	0.381	1.06 (0.90–1.25)	0.480
#Small for date (*N* = 10,804)	127 (3.8%)	238 (3.2%)	1.20 (0.97–1.50)	0.091	1.17 (0.92–1.47)	0.194
Major congenital anomalies (*N* = 10,828)	70 (2.1%)	120 (1.6%)	1.32 (0.98–1.77)	0.070	1.37 (1.00–1.86.00.86)	0.049
Chromosomal anomalies	13 (0.39%)	18 (0.24%)	1.62 (0.76–3.32)	0.184	1.56 (0.75–3.28)	0.236
#Stillbirth (*N* = 10,804)	15 (0.45%)	14 (0.19%)	2.41 (1.16–5.00.16.00)	0.018	2.58 (1.19–5.61)	0.016
#Stillbirth in babies without congenital anomalies (*N* = 10,632)	14 (0.43%)	12 (0.16%)	2.63 (1.22–5.71)	0.014	3.10 (1.36–7.02)	0.007
#Neonatal death (*N* = 10,727)	15 (0.45%)	17 (0.23%)	1.99 (0.99–3.99)	0.053	2.10 (1.00–4.36.00.36)	0.049
#Neonatal deaths in babies without congenital anomalies (*N* = 10,598)	8 (0.24%)	6 (0.08%)	3.02 (1.05–8.70)	0.041	3.15 (1.04–9.52)	0.042

Gestational diabetes adjusted for age, BMI categories nationality, parity, assisted reproduction, thyroid disease, chronic hypertension

Gestational hypertension adjusted for age, BMI categories, nationality, parity, preexisting diabetes, assisted reproduction

Multiple gestation- adjusted for age, BMI categories, nationality, parity and assisted reproduction

Preterm births- adjusted for age, parity, BMI categories, preexisting medical disorders and assisted reproduction

Low birth weight- adjusted for age, parity, BMI categories, nationality, preexisting disorders and assisted reproduction

Congenital anomalies adjusted for age, nationality, BMI, assisted reproduction, preexisting disorders

Chromosomal anomalies adjusted for age, nationality, BMI, preexisting disorders

Stillbirth and neonatal death adjusted for age nationality, parity, BMI, assisted reproduction, preexisting medical disorders

*OR *odds ratios; *CI *confidence intervals; *p* < 0.05 considered evidence against null hypothesis; #- only pregnancies ≥ 24 weeks gestation included; *CU* consanguineous unions, *NCU *non-consanguineous unions

In pregnancies ≥ 24 weeks GA, the risk of stillbirth in the cohort was 2.6 per 1000 births (4.5 in CU vs. 1.9 in NCU). The incidence was higher in first-degree (4.8 per 1000 births) compared to 3.4 per 1000 in second-degree (Figs. 1, 2 and 3). The odds of stillbirth were 2.58 times higher in CU compared to NCU (95% CI = 1.19–5.61; *p* = 0.016). Even in structurally normal babies, CUs had 3.10 times higher odds (95% CI = 1.36–7.02; *p* = 0.007) after adjusting for confounders. Similarly, the incidence of neonatal deaths in viable pregnancies was 3 per 1000 births (4.5 per 1000 in CU vs. 2.3 per 1000 in NCU). The incidence was similar among those with first-degree and second-degree CU. After adjusting for confounders, those pregnancies in CU had 2.10 times higher odds of neonatal death (95% CI = 1.00–4.36.00.36; *p* = 0.049) and 3.15 times higher odds of neonatal deaths even in structurally normal babies (95% CI = 1.04–9.52; *p* = 0.042).

Higher-order pregnancies were 50% less in CU compared to NCU (aOR = 0.47, 95% CI = 0.31–0.71; *p* < 0.001). The incidence of chromosomal anomalies was 2.8 per 1000 births, increasing to 4.8 per 1000 with first-degree consanguinity (vs. 1.1 per 1000 in second-degree). The adjusted odds in CU were 56% higher than NCU; however, this association did not reach statistical significance (aOR = 1.56, 95% CI = 0.75–3.28;*p* = 0.236). There was no statistically significant association between consanguinity and GDM, PIH or PTB. Similarly, consanguinity did not significantly impact the risk of SGA or LBW babies.

## Discussion

This study highlights the prevalence of consanguinity in the pregnant population in Qatar, with 46% of nationals and 25% of expatriates having CUs, two-thirds of which are first-cousin marriages. We report significantly higher odds of major congenital anomalies, stillbirths, and neonatal deaths in non-anomalous babies in pregnancies from these unions. The risks were higher in first-degree CUs, while second-degree and NCUs had comparable risks. The incidence of neonatal complications associated with CUs in the multinational expatriate population has been reported in this study for the first time, with Qatari women having a different risk profile when compared to non-Qatari.

Consanguinity has been occurring worldwide, dating back to the early years of civilization, as a way to propagate the species. As the species expanded and migrated worldwide, the rates have steadily decreased, and currently, only 10% of the global population consist of CU couples and their children. A horizontal geographical area passing across North Africa, the Arab world including Middle-Eastern countries, and Central and South Asia have CU rates ranging between 20 and 50% of the population [2, 20], often due to cultural, social, economic and political reasons. In Qatar, a population study in 2004 reported a rate of 54% in Qatari women, with CU rates higher in the index generation compared to the previous generation [10]. This could be attributed to the increased longevity and survival into adulthood, resulting in increased availability of marital options within the family [2].

In this study, we report an overall CU rate of 31%, two-thirds being first-degree. In Qatari women, 46% are CUs, similar to previous reports [10, 11]. The distribution of first and second-degree consanguinity are also similar, providing more validity to our results. In contrast, 25% of the expats have CU, with a slightly higher rate of second-degree than the nationals. The expats in Qatar mainly consist of women from other Arab countries and Southeast Asia, namely India and Pakistan, where consanguinity is also customary, which is reflected in the higher-than-global CU prevalence.

**Fig. 4:**
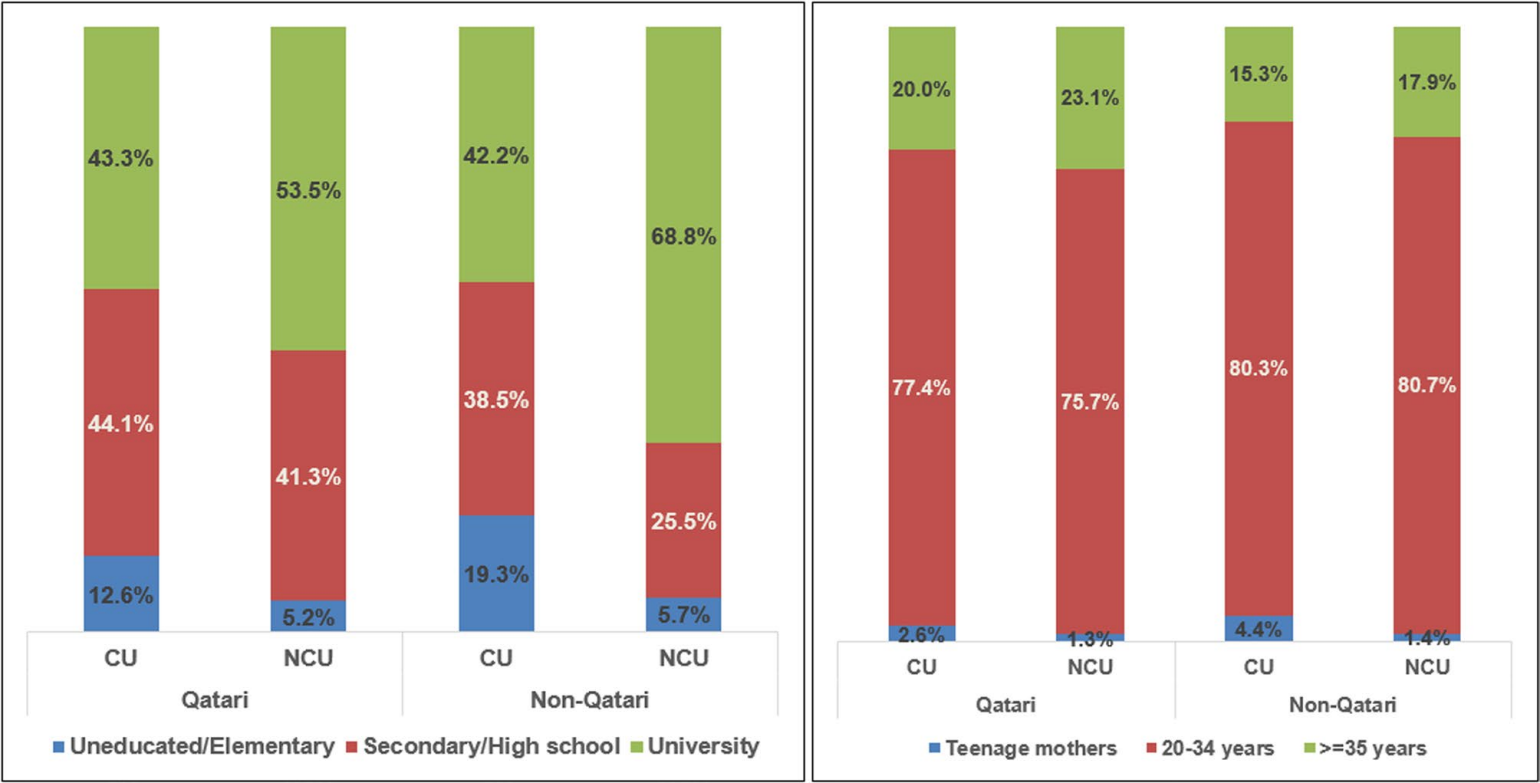
Distribution of consanguinity groups in Qatari and Non-Qatari women according to education level and maternal age; CU- consanguineous unions, NCU- non-consanguineous unions

The similarity of our results to numbers published more than a decade ago demonstrates the persistence of the drivers of CU within the country. The custom is generally due to cultural and economic reasons, with the retention of family property considered a significant advantage [10]. Other considerations include better relationships with the in-laws as women will be considered a part of the extended family and better support and care for the offspring [1]. A systematic review focussing on drivers of CU in the Arab world reported an association with younger age groups (especially teenage mothers) in all populations included [20], as reported in our study (Fig. 4). The earlier marriages explain the increased parity in CUs as well. The review also reports low maternal educational levels as another important driver, reflecting traditions that prefer early marriages within the family over education. In our study, the contrast between CU and NCU is seen more in the expats (Fig. 4), whereas, in the nationals, the contrast is much less, with similar numbers in both groups completing university education. This emphasizes that local customs are stronger among the nationals as more than 43% of well-educated women enter CUs as well. Previous studies have shown that maternal education is not a risk factor for outcomes such as stillbirth or anomalies independent of consanguinity [4], which rationalizes our choice of not including maternal education as a confounder.

There has been a growing debate about whether the socioeconomic advantages of consanguinity outweigh the risks. A cross-sectional review of the knowledge and attitudes towards consanguinity among the Qatari population demonstrated that the majority felt marrying into the extended family is always beneficial due to familiarity, with the added advantage of maintaining traditions and better childcare [12]. However, the concerning fact was that a major portion of those who came from families where CUs occurred were not aware of the associated genetic and health consequences. The past few decades have witnessed commendable advancements in the health care system of Qatar, curbing the mortality and morbidity from infectious diseases and malnutrition. But the burden from genetic illnesses is still on the rise. Our study will help highlight the impact of consanguinity on perinatal health and spread awareness, ultimately improving healthcare provision.

We report associations between CUs and congenital anomalies, stillbirth and neonatal death in this study, all of which corroborate well with previous studies. A large prospective Lancet study conducted in England demonstrates a doubling of the risk of congenital anomalies in CUs, mainly in the Pakistani population [21]. They report a 67% higher rate of anomalies, which can be explained by the exclusion of pregnancies < 20 weeks in our study. Studies from Middle Eastern countries report similar associations, especially CVS anomalies in first-degree CU, as demonstrated in our study [1, 9, 22]. In contrast, CNS anomalies are commoner in the Non-Qatari CUs in our cohort. This association has been observed in studies from other Arab countries but not those from the Middle East with a genetic profile similar to the Qatari population [23–25]. The results of this study including women from different nationalities can help in prenatal counselling in other parts of the world where the population is becoming increasingly multiethnic due to widespread emigration.

A cross-sectional study of 600 families in Qatar reported higher odds of autosomal recessive disorders in CUs and much higher odds of chromosomal anomalies in NCUs [13]. The exclusion of early trimester miscarriages could explain why we could not detect this association in this study. A case-control study looking at the risk factors for anomalies in live births in Qatar could not show a significant association with CUs [26], probably due to differences in study design, population and the variables adjusted for.

There has been conflicting evidence on the effect of consanguinity on stillbirth and neonatal death. Many studies from Australia, Sweden and India report an association [27–29]; however, no significant association was noted for fetal loss in studies from Arab countries such as Saudi Arabia, Sudan and Jordan [30–32]. A study of 1,800 Qatari women reported slightly higher live births in CUs than NCUs [11]. This contrasts with our results because a broader population, including expatriates, are evaluated in this study, which could explain the higher fetal loss rates. Additionally, the previous studies neither stratified the results according to the degree of consanguinity nor tried to explore if the association was significant in morphologically normal babies, which is a novelty in our study. Although there are reports of increased preterm birth in CUs [33], we were not able to detect this association.

Over the past decade, Qatar has successfully established effective genetic counselling services, including premarital and prenatal counselling [34]. Premarital testing for recessive genes is required for Qatari couples as per current government regulations in order to prepare the couple for possible outcomes. The premarital program, started in 2009, has been quite effective in reducing the incidence of conditions such as cystic fibrosis, homocystinuria and spinal muscular atrophy [35]. The screening programs, however, are not mandatory for other nationalities; therefore the incidence of adverse outcomes in CU couples persists in non-nationals, partially explaining the results seen in our study.

There has been an increase in genetic research in the country to understand the genetic makeup of the national population through initiatives such as Qatar Biobank and Qatar Genomic Project, and make progress in precision medicine [36]. Such advances could possibly lead to solutions to tackle these consequences in this country, where CUs are acceptable and desired. Despite the advances, social, cultural and religious barriers still exist, and many CU couples fail to access these services and are reluctant to consider counselling. It would be essential to disseminate the results via government social media platforms to ensure widespread dissemination of information. Increased awareness about the consequences on consanguinity among all families (citizens and residents) is important to help improve the genetic constitution in future generations, by encouraging couples to attend genetic counselling services and ensuring that the pregnancies are managed by a qualified multidisciplinary team.

### Strengths and limitations

To the best of our knowledge, this is the first large scale population-based study from Qatar exploring the impact of CUs on pregnancy and neonatal outcomes in both nationals and expats. The large sample size, the biggest strength of the study, included a heterogeneous population from almost 95 different countries and is a more accurate representation of the maternity population in Qatar. This study also quantifies the associations between CU and various maternal demographics, providing more insights into the possible factors associated with the higher rates of CU in the population.

Some limitations need to be kept in mind while interpreting the results. The analysis has been restricted to those women with information regarding consanguinity documented in their medical records. To mitigate this potential bias, we have included a large sample that is representative of the population. Miscarriages less than 20 weeks’ gestation were not included in the study, hence the risk of congenital and chromosomal anomalies reported in this study can be applied only to babies surviving beyond 20 weeks. Previous reports suggest that at least 50–60% of first trimester and nearly 24% of second trimester losses can be attributed to congenital anomalies and chromosomal abnormalities [37], with nearly two times higher odds in CU as mentioned previously. The true impact of CU on these outcomes might be underreported in our study, as the exclusion of < 20 weeks pregnancies most likely biased the results towards the null. The exposure variable (consanguinity status) is self-reported by the women and the family histories has not been cross-verified. However, considering the marriage laws in the country, the possible associated misclassification is more likely to underestimate consanguinity and shift the results towards the null. Although the associations have been adjusted, residual confounding is still a possibility, especially for stillbirths and neonatal deaths.

## Conclusion

Our study establishes that consanguinity is associated with a multitude of complications including increased incidence of major congenital anomalies, stillbirth, and neonatal deaths. The detrimental effects were more pronounced in first-degree CUs compared to second-degree. This information needs to be disseminated in the public, which will help CU couples approach genetic counselling services in the country. The genetic clinics are linked already to high-risk fetal maternal surveillance units in order to ensure the best care for affected pregnancies. These pregnancies should ideally be managed by a multidisciplinary team including consultant obstetrician, foetal medicine specialist, geneticist and patient and family educator. The advances in genetic research and genome mapping could possibly find solutions for the adverse perinatal outcomes associated with consanguinity, especially in populations where CUs are considered a socio-economic advantage.

## Data Availability

The datasets generated and/or analysed during this study are not publicly available due to participant confidentiality concerns and institution guidelines. However, they can be accessed from the corresponding authors on reasonable request, after seeking approval from the participating institution.

## Abbreviations

aOR: Adjusted odds ratios
ART: Assisted reproduction techniques
BMI: Body mass index
CI: Confidence intervals
CNS: Central nervous system
CU: Consanguineous unions
CVS: Cardiovascular system
GA: Gestational age
GDM: Gestational diabetes mellitus
IQR: Inter quartile range
LBW: Low birth weight
MRC: Medical Research Council
NCU: Non-consanguineous unions
ND: Neonatal deaths
NICU: Neonatal intensive care unit
PIH: Pregnancy-induced hypertension
PTB: Preterm birth
SD: Standard deviation
SGA: Small for gestational age
WHO: World Health Organization
WWRC: Women’s Wellness and Research Centre
